# Association of Per- and Polyfluoroalkyl Substances with Allostatic Load Stratified by Herpes Simplex Virus 1 and 2 Exposure

**DOI:** 10.3390/toxics11090745

**Published:** 2023-09-01

**Authors:** Yvonne S. Boafo, Sayed Mostafa, Emmanuel Obeng-Gyasi

**Affiliations:** 1 Department of Built Environment, North Carolina A&T State University, Greensboro, NC 27411, USA; 2 Environmental Health and Disease Laboratory, North Carolina A&T State University, Greensboro, NC 27411, USA; 3 Department of Mathematics and Statistics, North Carolina A&T State University, Greensboro, NC 27411, USA

**Keywords:** herpesvirus 1 & 2, human, allostatic load, perfluoroalkyl substances

## Abstract

Herpes Simplex Virus (HSV) 1 and 2 are persistent infections that affect a significant percentage of United States (US) adults, with 48% having HSV-1 and 12% having HSV-2. Using data stratified by HSV-1 and HSV-2 exposures, this study investigated the association of per- and polyfluoroalkyl substances (PFAS), a group of toxic synthetic organofluorine chemical compounds found in environmental, occupational, and home settings, with allostatic load (AL), an index of chronic physiological stress. Descriptive statistics, multivariable logistic regression, and Bayesian Kernel Machine Regression (BKMR) modeling were used to assess the effects of multi-PFAS exposures on AL using data from the National Health and Nutrition Examination Survey (NHANES) 2007–2014. Results indicated participants not exposed to PFAS exhibited 77% to 97% lower odds of higher AL (*p* < 0.001). For example, PFOS per unit increase brought forth a 2% odds increase in higher AL (OR: 1.02; 95% CI: 1.00, 1.05; *p* < 0.05). Participants exposed to PFAS had reduced odds of higher AL (77%–79%), regardless of their HSV-1 and HSV-2 status. PFAS exposure was more prevalent in those with HSV-1 (60%) than in those with HSV-2 (20%) infection, while AL levels were comparable in both groups (17%). BKMR revealed a nonlinear PFAS-AL association and confirmed interactions among PFAS. In summary, PFAS exposure increased the likelihood of higher AL among those with persistent HSV infections. Our study enhances the current understanding of the complex dynamics involving PFAS, persistent infections, and AL, which hold significant implications for public health and clinical intervention strategies.

## 1. Introduction

Per- and polyfluoroalkyl substances (PFAS), a group of synthetic chemicals, find widespread usage in industrial, commercial, and consumer products, involved in the manufacturing of various items like lubricants, paints, food packaging, fire-extinguishing foams, non-stick coatings on cookware, and more [1]. They can be found in the air, soil, water, and contaminated food [2] and are hazardous to humans and animals. Given their ubiquitous presence in the environment and potential adverse impacts on human health and wildlife, PFAS have garnered significant public health interest and prompted extensive scientific research on the health effects linked to PFAS exposure [2].

Recent studies have highlighted the association between PFAS exposure and prenatal and fetal growth dysfunction [3], underscoring its early-life effects. Persistent exposure to PFAS leads to a cascade of adverse health effects, affecting multiple systems within the human body and bringing forth extensive health consequences. These consequences include elevated lipid levels, hepatic dysfunction, compromised immune function, increased risk of hypertension, insulin dysregulation, kidney disease, and cancer [2,4]. PFAS has unique properties that make them persistent in the environment and resistant to degradation, leading to their accumulation in soil, water, and various organisms. Since PFAS are not easily eliminated from the body, repeated exposure to these substances can result in higher concentrations over time, leading to potential health concerns [5]. These findings underscore the critical importance of comprehensively examining the consequences of PFAS exposure and its potential to inflict long-term harm on human health. This study focuses on perfluorooctanoic acid (PFOA), perfluoro octane sulfonate (PFOS), perfluoro hexane sulfonate (PFHxS), and perfluoro nonanoic acid (PFNA), as they are more prevalent and widely studied, and PFOA and PFOS are abundantly produced in the U.S. [2].

AL refers to the cumulative physiological burden experienced by an individual as they adapt to prolonged exposure to various stressors. These stressors can trigger neuroendocrine responses, leading to body system adjustments to maintain stability and cope with the challenges. Over time, the repeated activation of these responses can lead to wear and tear on the body, potentially increasing the risk of various health problems [6]. AL relies on a complex interplay of social, physical, and environmental factors, which intricately shape an individual’s behavioral responses and systemic physiology [6,7,8], leading to aberrant alterations in normal physiological processes and maladaptation of crucial health markers [9]. Ultimately, the wear and tear over time [6,10] owing to AL [11,12,13] leads to weathering of the body and adverse health outcomes [10]. As such, AL serves as a mediator for several chronic diseases by acting as a crucial link between chronic stress and adverse health outcomes. The wear and tear experienced by the body due to prolonged and repeated activations of the stress response systems can contribute to the development and progression of various health conditions.

Chronic diseases such as cardiovascular disorders, metabolic syndrome, immune dysregulation, and mental health disorders have been associated with elevated AL. The continuous strain on physiological systems and organs can lead to inflammation, hormonal imbalances, impaired immune function, and altered metabolism, creating a favorable environment for chronic disease development.

Moreover, AL has been implicated in exacerbating pre-existing health conditions and influencing the severity and prognosis of illnesses [14,15].

Persistent infections can involve various microorganisms, such as viruses, bacteria, parasites, and fungi. These infections persist within specific cells of the infected host over an extended period, often transitioning from a silent to a productive stage without causing rapid damage to the host cell. As a result, the host’s infection can manifest as latent, chronic, or slow. Factors such as physical stress, trauma, other conditions, or fluctuations in cell physiology can initiate latent infections. The consequences of these persistent infections can lead to various diseases [16]. Latency is a characteristic feature of viruses in the Herpesviridae family, where the virus remains dormant within the host’s cells. During this latent phase, the virus does not actively replicate or cause significant harm to the host. However, specific triggers can reactivate the virus, leading to a productive or lytic phase, during which the virus starts replicating and causing symptoms.

Various factors, including stress-related stimuli, adrenal hormones, immunosuppression, and emotional disturbances, can trigger the reactivation of latent herpesviruses. These triggers can disrupt the delicate balance that maintains the virus in its latent state, leading to the resumption of viral replication and the potential to develop clinical symptoms [9]. HSV-1 and HSV-2 viruses can cause considerable discomfort and psychological stress, significantly impacting overall well-being. HSV-1 is responsible for cold sores and establishes a chronic infection. On the other hand, HSV-2 is primarily transmitted through sexual contact and is considered a sexually transmitted infection (STI). Both viruses can significantly affect individuals’ physical and emotional health [17]. As such, they are ideal for examining the effects of PFAS and persistent infections on AL. HSV-1 and HSV-2 are persistent infections that affect a significant percentage of United States (US) adults, with 48% having HSV-1 and 12% having HSV-2 [18].

It is crucial to study the relationship between PFAS and AL among those with and without persistent infections to understand the effects of PFAS on AL in the physiological environment brought forth by these infections [1]. Studies show a growing interest in understanding the combined effect of environmental mixtures and stressors and the consequences of these factors when experienced in combination [19]. Still, a gap exists in the literature regarding these relationships in the presence of persistent infections such as HSV-1 and HSV-2. This study seeks to close the gap in the literature on this crucial area of inquiry.

This study’s overarching hypothesis posits that individuals exposed to persistent infections exhibit a significant association between PFAS exposure and AL.

This study’s primary objective is to explore the relationship between PFAS exposures (PFOA, PFOS, PFNA, and PFHxS) and AL stratified by the status of HSV-1 and HSV-2.

The study adjusts for covariates such as age, gender, race, education, household income, and marital status. The covariates were chosen based on the literature suggesting their relationship with the variables of interest [6,8,20].

## 2. Materials and Methods

### 2.1. Description of the Study Participants

The NHANES is a recurring cross-sectional survey conducted by the National Center for Health Statistics (NCHS) biennially since 1999. This study uses four cycles of NHANES data from 2007 to 2014 to obtain sample data representative of the U.S. population. This includes the 2007–2008, 2009–2010, 2011–2012, and 2013–2014 cycles. The NHANES employs stratified, multistage probability sampling methods. It serves as a valuable tool to obtain national estimates of the demographic, health, and nutritional indicator status of the civilian, noninstitutionalized population of the United States [21]. Households are invited to participate in the survey by email and complete an online questionnaire to determine eligibility. Eligible individuals are contacted for phone interviews at agreed-upon times and then scheduled for health exams at the NHANES Mobile Exam Center [22]. Participants provided written consent to participate in the survey and were informed that the data collected would not be used without their permission. Indeed, the informed consent indicated that the collected data would be used for the stated purpose, as illustrated by Section 308(d) of the Public Health Service Act (42 U.S.C. 242 m). The National Center for Health Statistics Ethics Review Board approved the data from 2007 to 2014 used for this study [23]. Our study did not include minors.

### 2.2. Quantifying PFAS and HSV

The CDC measures 12 PFAS concentrations, including PFOA, PFOS, PFNA, and PFHxS, in the serum of NHANES survey participants aged 12 and older. Detailed laboratory procedures are on the CDC’s website [24]. Regarding HSV-1 and HSV-2, antibodies to HSV-1 and HSV-2 were assessed using validated CDC laboratory methods [25]. 

### 2.3. Operationalizing Allostatic Load

Researchers conceptualized AL as a cumulative indicator of overall wear-and-tear on the body in response to adapting to environmental stresses [9,10]. AL is used as an antecedent for chronic diseases [9]. The study used the following biomarkers to operationalize AL: total cholesterol (TC), high-density lipoprotein (HDL) cholesterol, glycosylated hemoglobin (HbA1c), albumin (Alb), triglycerides (TG), body mass index (BMI), creatinine clearance (CLCR), C-reactive protein (CRP), systolic blood pressure (SBP), and diastolic blood pressure (DBP). This is consistent with previous studies that have used biomarkers from cardiovascular, metabolic, and immune systems [9,14]. The biomarkers were transformed into quantiles based on the data distribution. The top 25% of the distribution for seven markers: C-reactive protein (CRP), triglycerides (TG), total cholesterol (TC), systolic blood pressure (SBP), diastolic blood pressure (DBP), body mass index, and glycosylated hemoglobin, were designated as high risk to determine higher AL. The bottom 25% of the distribution of albumin, creatinine clearance, and HDL cholesterol were also considered high-risk, as a lower value for these markers indicated dysfunction. In forming the index out of 10 for everyone within the study, all high-risk individuals, for each marker were assigned a 1, with non-high-risk individuals given a 0 to obtain a total AL value. An AL value greater than or equal to 3/10 was considered elevated. 

### 2.4. Statistical Analysis

#### 2.4.1. Descriptive Statistics

Descriptive statistics are presented to describe the distribution of the exposure and demographic variables in the dataset and stratify them by the response variable (AL). Spearman correlation was used to assess the relationships among the PFAS exposure variables. To test for differences between participants with and without elevated AL, we used the chi-square test for categorical variables and the Wilcoxon test for continuous variables. 

#### 2.4.2. Multivariable Logistic Regression

The study used multivariable logistic regression analysis to analyze the association between combined exposures to PFAS components PFOA, PFOS, PFNA, and PFHxS and the higher AL. Binary AL (non-elevated = 0 vs. elevated = 1) was the response variable in all the logistic regression models. Three models were fitted using the continuous values and the categorical variables, and estimated odds ratios and corresponding 95% confidence intervals were obtained. The fitted models included unadjusted PFOA, PFOS, PFNA, and PFHxS for participants who tested positive and negative for HSV-1 and HSV-2 separately. The second group of models included PFOA, PFOS, PFNA, and PFHxS adjusted for age, gender, race/ethnicity, education level, household income, and marital status for participants who tested positive or negative for HSV-1 and HSV-2 separately. The third group of models accounted for the interaction between the PFAS components. 

#### 2.4.3. Bayesian Kernel Machine Regression 

BKMR modeling assessed the combined effects of PFOA, PFOS, PFNA, and PFHxS on AL stratified by HSV-1 and HSV-2. Many researchers have used BKMR to determine the cumulative effect of exposure to multi-pollutant mixtures [1,2,3,4,5,6] on diverse health outcomes, including obesity [7], diabetes [1,3,5], and cardiovascular disease [20], owing to the technique’s ability to model non-linear and non-additive relationships [19,26] and give a more accurate assessment of the combined effect of contaminants on an outcome of interest.

Denoting the binary response variable by Y, the exposure variables by $z_{1\;},z_{2},\ldots ,\;z_{M}$, and the vector of covariates by x, the BKMR model can be expressed as follows:

$$g\left(\mu_{i}\right)=h\left(z_{i1,}\ldots \ldots .,\;z_{iM}\right)+{\beta x}_{i};\;i=1,\;\ldots .,\;n,$$

where g is a monotonic link function on $\mu_{i}=E(Y_{i})$, h is a flexible function of the exposures, and β is a vector of coefficients for the covariates that are assumed to be linearly related with the response variable [26]. The functional form of the function h is determined by a kernel function K. The Gaussian kernel is a common choice as it can capture various functional forms for h. With this choice of the kernel function, the following representation of h is implied:

$$K\left(z_{i},z_{j}\right)=\exp\left\{-\sum_{m=1}^{M}r_{m}{\left(z_{im}-z_{jm}\right)}^{2}\right\}$$

where $z_{i}$ and $z_{j}$ are vectors of exposures for two individuals, and the weight $r_{m}\geq 0$ represent the probability that the m-th exposure is important in the function h. As such, $r_{m}=0$ would indicate that the m-th exposure has no significant contribution to h and thus has no significant association with the response. $r_{m}$ can also be viewed as a smoothing parameter that controls the smoothness of h as a function of the exposure $z_{m}$ [26].

In this study, the predictors (exposures), Z, are the PFAS variables, and the Gaussian kernel represents h. The BKMR models exposure via a kernel function [19,26] to explore the exposure-response association considering the non-linear and non-additive complex relationships between the PFAS components and AL [19,26]. The Markov chain Monte Carlo method is commonly used to fit the BKMR model and estimate the posterior inclusion probability (PIP), which measures variable importance and helps select the most critical exposures [6]. The BKMR model uses multiple representations to explore patterns of association and interaction between PFAS and AL. The process provides an understanding of univariate, bivariate, and trivariate associations, interactions, overall effects, single-variable effects, and single-variable interactions [19,27]. This approach helps to account for the complex effects of exposure to environmental mixtures using multiple scenarios of exposure-response functions [19,26,27].

This study conducted all analyses using the statistical package R (version 4.2.1; R Foundation for Statistical Computing, Vienna, Austria). A *p*-value < 0.05 was considered statistically significant.

## 3. Results

### 3.1. Characteristics of the Sample Participants

One thousand seven hundred and eighty-four (1784) participants aged 20 to 49 met the inclusion criteria for our study. All these participants were exposed to PFOA, PFOS, PFNA, and PFHxS and tested for HSV-1 and 2. None of the participants had a missing value for any of the covariates of interest, including age, gender, education, race, household income, or marital status. Overall, the prevalence of HSV-1 was high at 60.59% compared to 19.99% for HSV-2. Among participants who tested positive for HSV-1, 17.23% experienced elevated AL. A similar percentage of elevated AL was observed among those who tested positive for HSV-2 (17.17%).

Males were likelier to have higher AL than females, as 19.08% of males had elevated AL, whereas 13.36% of females had elevated AL. The result was significant (*p* = 0.005). Overall, individuals with a higher AL tended to be older than those with a lower AL (37 years old compared to 32.95 years old). Individuals with lower education constituted a more significant proportion of those with higher AL than their more educated counterparts. Participants with household incomes of less than USD 55,000 were more likely to experience elevated AL than those with USD 55,000 or more. Specifically, 17.28% of participants with household incomes below USD 25,000 had elevated AL, compared to 9.90% of those with household incomes of USD 75,000 and above. The data shows that married individuals and those living with partners were more likely to experience elevated AL at 18.18% compared to single individuals at 13.63%. This result was significant, with *p* = 0.02. Table 1 and Table 2 below summarize the findings for the total population and the population stratified by AL status, respectively.

### 3.2. Correlations between PFAS Exposures

**Figure 1:**
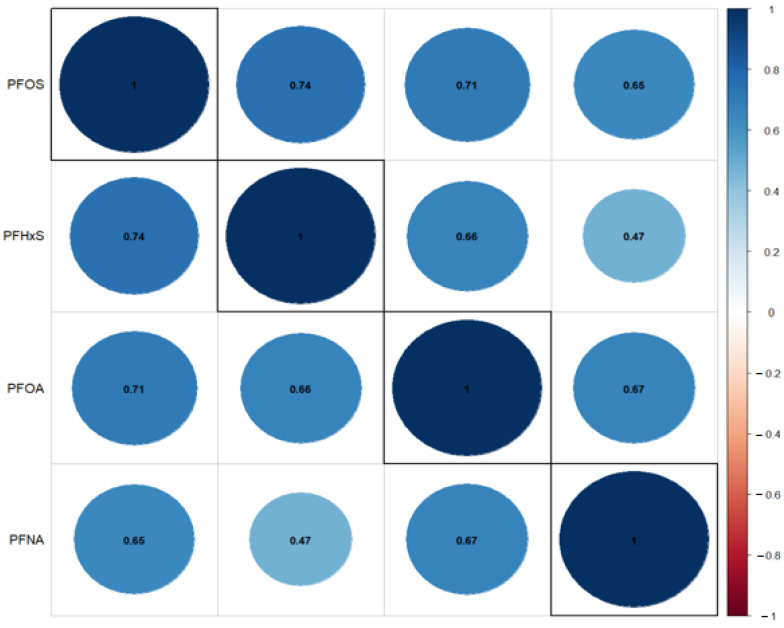
Correlation Matrix between pairs of PFAS components—PFOA, PFOS, PFNA, and PFHxS. *p*-values for the above correlation coefficients are all <0.001 threshold and are highly significant.

We performed correlation analysis using the Spearman method to measure the strength and direction of the monotonic or nonlinear relationship between PFOA, PFOS, PFNA, and PFHxS. The chart below shows a strong positive correlation between PFOA, PFOS, PFNA, and PFHxS pairs, with a correlation coefficient ranging from 0.47 between PFOA and PFHxS to 0.74 between PFOS and PFHxs. This means that as PFNA levels go up, PFHxs levels tend to go up, and as PFNA levels go up, PFHxS levels increase. See the result below, which highlights the most important and critical relationships. The correlation between PFOA and PFOS was *r*(1782) = 0.7, *p* < 0.05, and PFOA and PFHxS was *r*(1782) = 0.66, *p* < 0.05. The results are presented in Figure 1 below.

### 3.3. Results of Logistic Regression

#### 3.3.1. Association between PFAS and AL Using Logistic Regression: Unadjusted for Covariates

The study used logistic regression models to explore the association between PFAS and AL among participants with positive and negative test results for HSV-1 and HSV-2. The unadjusted models showed that participants were less likely to experience elevated AL when not exposed to PFOA, PFOS, PFNA, and PFHxS. The results for participants who tested positive for HSV-1 were OR: 0.21 (95% CI: 0.18, 0.24; *p* < 0.001). Outcomes for participants who tested negative for HSV-1 were OR: 0.23 (95% CI: 0.15, 0.37; *p* < 0.001). Among participants who tested positive for HSV-2, the results were OR: 0.23 (95% CI: 0.14, 0.39; *p* < 0.001), and for participants who tested negative for HSV-2, the observed effects were OR: 0.21 (95% CI: 0.15, 0.28; *p* < 0.001). The results revealed that participants had between 77% and 79% reduced odds of experiencing higher AL without exposure to PFOA, PFOS, PFNA, and PFHxS, irrespective of test results for HSV-1 or HSV-2. The results indicated that among those who tested negative for HSV-1, individuals exposed to PFOS were more likely to have a higher AL. In contrast, those exposed to PFNA were less likely to have a higher AL.

Moreover, for model 2, among respondents who tested negative for HSV-1, in addition to the intercept, the observed result indicated a one-unit increase in exposure to PFOS was associated with a 2% increase in the odds of higher AL, with observed results of OR: 1.02 (95% CI: 1.00, 1.05, *p* = 0.035), statistically significant at the 0.05 threshold.

Additionally, exposure to PFNA was associated with 40% decreased odds of higher AL, with observed results of OR: 0.60 (95% CI: 0.42, 0.86; *p* = 0.006), statistically significant at the 0.05 threshold. Table 3 below summarizes the results.

#### 3.3.2. Association between PFAS and AL by HSV Exposure Status, Adjusted for Study Covariates

The observed results show that the intercept models are statistically significant for all four adjusted models fitted to explore the association of PFOA, PFOS, PFNA, and PFHxS with AL, stratified by HSV-1 and HSV-2. The intercept models are significant for all four scenarios where participants are exposed to PFOA, PFOS, PFNA, and PFHxS, stratified by the test results for HSV-1 and HSV-2. The study participants were less likely to experience higher AL without exposure to PFAS. Specifically, the observed effects for participants who tested positive for HSV-1 were OR: 0.03 (95% CI: 0.01, 0.08; *p* < 0.001), OR: 0.23 (95% CI: 0.15, 0.37; *p* < 0.001) regarding participants who tested negative for HSV-1, OR: 0.23 (95% CI: 0.14, 0.39; *p* < 0.001) for participants who tested positive for HSV-2, and OR: 0.21 (95% CI: 0.15, 0.28; *p* < 0.001) for participants who tested negative for HSV-2. This indicates that participants have between 79% and 97% reduced odds of experiencing elevated AL without any PFAS components, irrespective of whether they tested positive or negative for HSV-1 or HSV-2. The models were adjusted for age, gender, race, education, household income, and marital status.

The intercept models for participants who tested positive for HSV-1 had a strong negative association with AL when they were not exposed to PFOA, PFOS, PFNA, and PFHxS. In addition, a unit increase in age produced 6% increased odds of higher AL, with observed results of OR: 1.06 (95% CI: 1.03, 1.08; *p* < 0.001) for this subgroup. Among participants who tested positive for HSV-1, being a male increased the odds of higher AL by 49%, with observed results of OR: 1.51 (95% CI: 1.07, 1.08; *p* = 0.021). Individuals with a household income of over USD 75,000 have 60% decreased odds of higher AL, with observed results of OR: 0.40 (95% CI: 0.18, 0.91; *p* = 0.029). Lastly, in this group, being non-married was associated with 46% decreased odds of AL, with observed OR results: 0.54 (95% CI: 0.34–0.85; *p* = 0.009). These models are statistically significant with values of <0.05.

Among participants who tested negative for HSV-1, PFOS has 2% increased odds of higher AL, with observed results of OR: 1.02 (95% CI: 1.00, 1.05; *p* = 0.035), while exposure to PFNA was associated with decreased odds of 40%, with observed values of OR: 0.60 (95% CI: 0.42, 0.86; *p* = 0.006). The results can be found in Table 4 below.

#### 3.3.3. Association between PFAS and AL, Accounting for Various PFAS Interactions

Among HSV-1-positive participants, similar to the adjusted model, lack of exposure to PFAS had a strong negative association with higher AL. The intercepts were significant, with *p*-values < 0.001. Those of older age and males had increased odds of elevated AL with higher household income, and being non-married was associated with decreased AL. This model accounted for PFOA, PFOS, PFNA, and PFHxS interactions. The logistic regression model shows no association between PFOA, PFOS, and PFNA’s joint effects, with an observed OR: 1.00 (95% CI: 1.00, 1.01, *p* = 0.034). However, the result demonstrated a bivariate interaction between PFOS and PFNA with values of OR: 0.97 (95% CI: 0.94, 1.00; *p* = 0.039), meaning the joint effect of exposure to PFOS and PFNA was associated with 3% decreased odds of higher AL, significant at the 0.05 threshold among participants who tested positive for HSV-1. Table 5 summarizes the association between PFAS and AL, accounting for the interaction between various PFAS.

### 3.4. Results of Bayesian Kernel Machine Regression 

#### 3.4.1. Variable Importance Results

Table 6 reports the PIP values from the BKMR models stratified by participants’ test results for HSV-1 and HSV-2. The PIP for predictor $z_{m}$ is the estimated probability of including the predictor in the model, or the proportion of MCMC iterations that are significant ${\;r}_{m}>0$. As noted earlier, the PIP indicates variable importance and helps rank critical variables in producing the outcome of interest [19,27].

Among participants who tested positive for HSV-1, PFHxS is more critical with a PIP of 0.4934, followed by PFOS (0.4700), PFOA (0.4659), and PFNA of 0.4604. None of the PFAS exposures stood out much more robustly than the others because of the slight difference between the values, and this could mean that PFOA, PFOS, PFNA, and PFHxS all have essential effects on AL.

Among participants who tested negative for HSV-1, PFNA was the most critical predictor explaining the difference in AL levels (the PIP for PFNA was 0.4853). PFOS ranked second again with a PIP of 0.4060. PFHxS (0.3486) and PFOA (0.3305) were less important among those who tested negative for HSV-1.

Among those who tested positive for HSV-2, PFOS with a PIP of 0.4526 was the most critical predictor of changes in AL levels, followed by PFHxS with a PIP of 0.4473. PFOA with a PIP of 0.4408 was slightly less important, and PFNA with a PIP of 0.3435 was the least significant predictor.

Among participants who tested negative for HSV-2, PFOS was the most important predictor of the difference in AL levels, with a PIP of 0.4732, followed by PFNA, which had a PIP of 0.4553. PFHxS was less critical with a PIP of 0.36072, and PFOA, with a PIP of 0.2970, had the least essential effect on changes in AL levels. 

Overall, PFHxS was an essential predictor of AL among participants with positive HSV, followed by PFOS and then PFOA. On the other hand, PFNA was a necessary predictor of AL among participants who tested negative for HSV-1 and the second most crucial predictor among those who tested negative for HSV-2.

Table 6 below shows the PIP for each PFAS component by herpes exposure. Darker colors correspond with the most critical components with a higher PIP, and lighter colors correspond with the least essential components with a lower PIP.

#### 3.4.2. Univariate Association of PFAS Components Stratified by HSV-1 and HSV-2 Test Results

**Figure 2:**
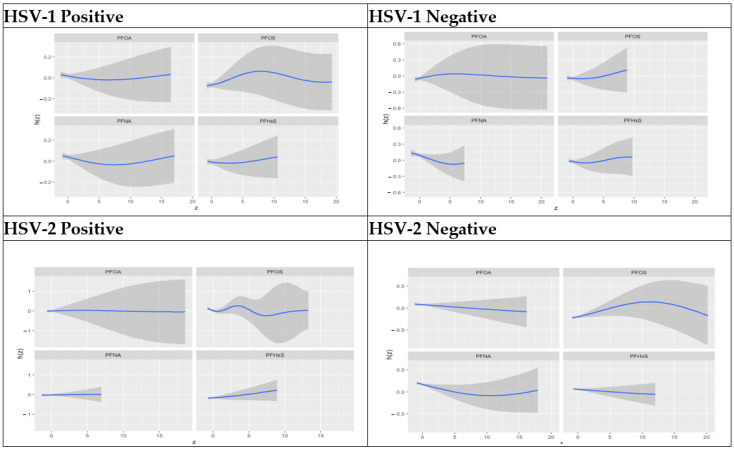
Univariate association of PFAS components stratified by HSV-1 and 2 test results.

The univariate approach visually examines the individual effects of PFOA, PFOS, PFNA, and PFHxS on AL. Figure 2 below shows the impact of an individual PFAS on AL when the other PFAS components are fixed at their median values. The gray bands represent 95% confidence intervals. The figure below shows the individual variables importance for higher AL and assesses the effect of HSV-1 and HSV-2 exposure on higher AL.

The above graph shows varying nonlinear associations between the predictors and AL. In all strata, PFOS has the most pronounced association with AL. For other exposures, the association is weak. All exposures show relatively wide confidence bands and thus relatively high uncertainties in the associations with AL.

#### 3.4.3. Bivariate Associations of PFAS Components Stratified by HSV-1 and HSV-2 Test Results

**Figure 3:**
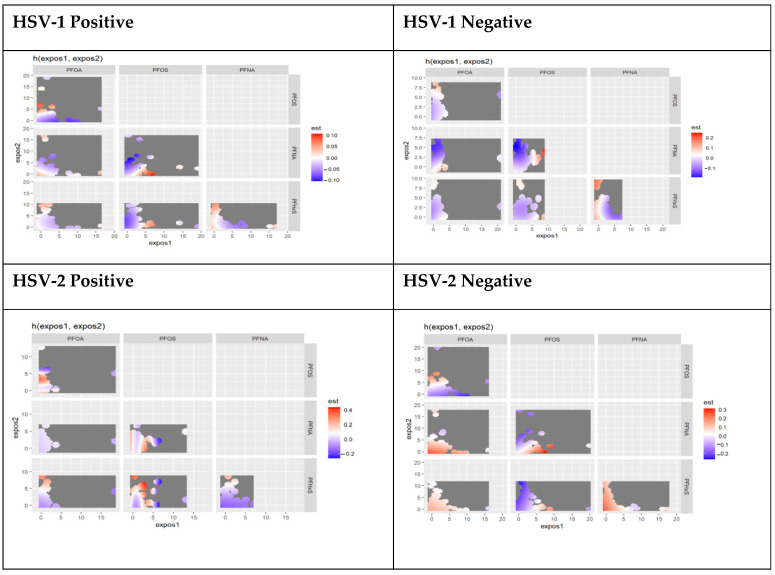
Bivariate association: bivariable exposure-response function of PFAS components stratified by HSV-1 and HSV-2 test results.

The results of the bivariate exposure-response function chart below show the interaction of the joint effect of pairs of PFAS components and AL with HSV results. The graph shows a negative-to-positive significant interaction between PFOA and PFOS and PFOS and PFNA for participants who tested positive for HSV-1. This confirms a nonlinear relationship between the joint effects of PFOA, PFOS, PFNA, PFHxS, and AL. The chart shows evidence of interaction between PFOS and PFHxs for participants who tested positive for HSV-2. For those who tested negative for HSV-2, there is a negative interaction between PFOS and PFHxS and AL and a negative to positive interaction between PFOS and PFNA with AL among this group. Figure 3 shows the interaction between the essential PFAS exposures based on exposure to HSV-1 and HSV-2.

#### 3.4.4. Interactions of PFAS Components Stratified by HSV-1 and HSV-2 Test Results

The joint effects of the PFOA, PFOS, PFNA, and PFHxS pairs are illustrated further by the bivariate association chart below. The chart examined the association between an individual PFAS component and AL fixing the second PFAS at different quantiles 25th (red line), 50th (green line), and 75th (blue line), with other PFAS held at the median. These models were adjusted for age, gender, race, education, household income, and marital status.

**Figure 4:**
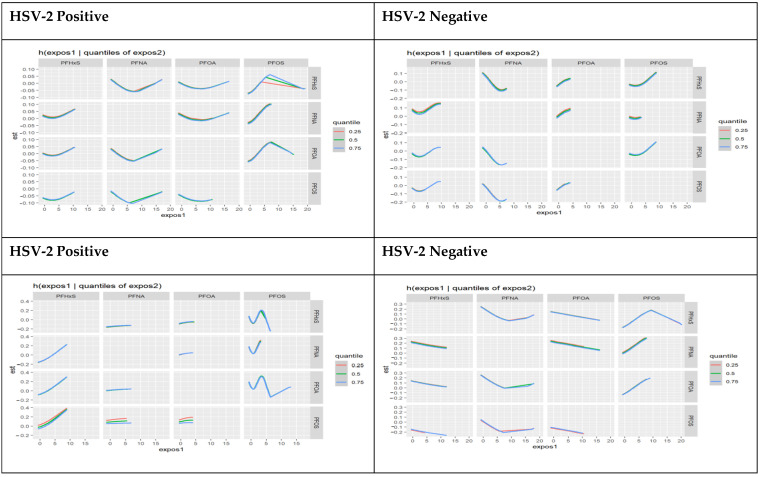
Bivariate association: interactions of PFAS components stratified by HSV-1 and HSV-2 test results.

The results show a strong interaction between PFOS and PFHxS, and the joint effect of the exchange is strong at 75% quantile compared to 25% quantile for participants who tested positive for HSV-1. Other components show some interaction, but the effect did not change significantly at different quantiles. Figure 4 below shows the results of the bivariate association between pairs of PFAS components at different quantiles.

#### 3.4.5. Three-Way Interactions of PFAS Components Stratified by HSV-1 and HSV-2 Test Results

**Figure 5:**
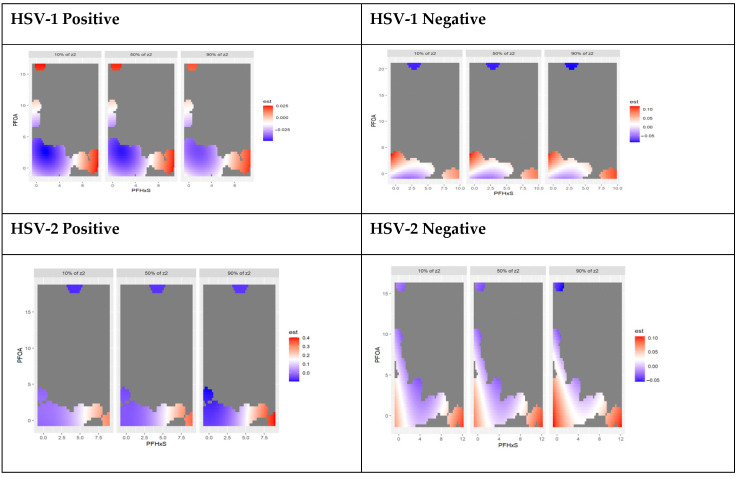
Three-way Interactions of PFAS components stratified by HSV-1 and HSV-2 test results.

The three-way interaction chart below (Figure 5) shows the effect of the association between PFOA and PFHxS when PFOS alternatives are from the 10th, 50th, and 90th percentiles. The chart shows a solid non-linear interaction for participants who tested positive for HSV-1 at all three quantile levels.

#### 3.4.6. Overall Exposure Effect of PFAS Components Stratified by HSV-1 and HSV-2 Test Results

**Figure 6:**
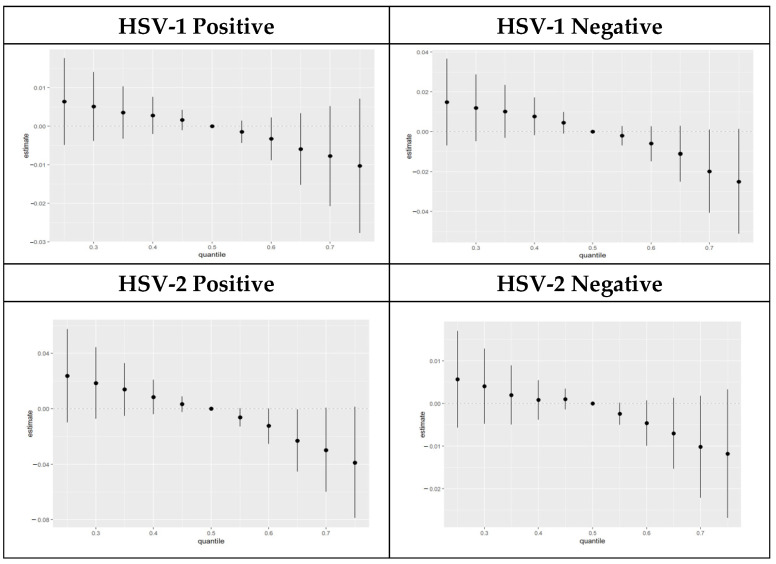
Chart showing the overall effect of PFOA, PFOS, PFNA, and PFNA at increasing quantiles.

Another interesting summary plot is the overall effect of the four PFAS exposures, calculated by comparing the value of h when all of the predictors are at a particular percentile to when all of them are at their 50th percentile. The charts below show that the joint effect of PFOA, PFOS, PFNA, and PFHxS decreases at higher quantile levels. Figure 6 below shows the overall result for all scenarios. PFHxS and PFOS were more critical for those who tested positive for HSV-1 and HSV-2; PFNA was more important for those who tested negative for HSV-1; and PFOS was more critical for those who tested negative for HSV-2. The result is that the slight difference in PIP for all four components is equally significant for this subgroup.

#### 3.4.7. Single Variable Effects of PFAS Components Stratified by HSV-1 and HSV-2 Test Result

**Figure 7:**
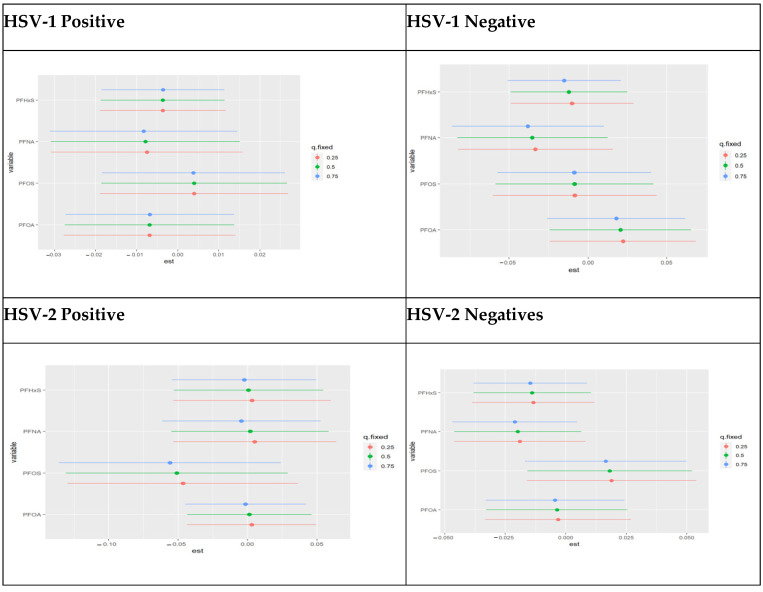
Chart showing the single variable effect of PFOA, PFOS, PFNA, and PFNA at increasing quantiles.

In Figure 7 below, the focus is on the individual PFAS exposure risk summaries at different quantiles on the 25th, 50th, and 75th. It considers the main effect and interaction effects of univariate PFAS components on the cumulative effect on AL as the individual components increase from the 25th to the 50th and 75th quantiles. Overall, there was little to no difference in the overall effects as the separate exposures alternated between the 25th, 50th, and 75th percentiles. Additionally, PFOS was positively associated with higher AL than PFNA among those who tested positive for HSV-1. In comparison, PFOA had a positive association with higher AL among those who tested negative for HSV-1. However, the difference is minimal since all four components are equally crucial for the various subgroups.

#### 3.4.8. Single Variable Interaction Terms of PFAS Components Stratified by HSV-1 and HSV-2 Test Results

**Figure 8:**
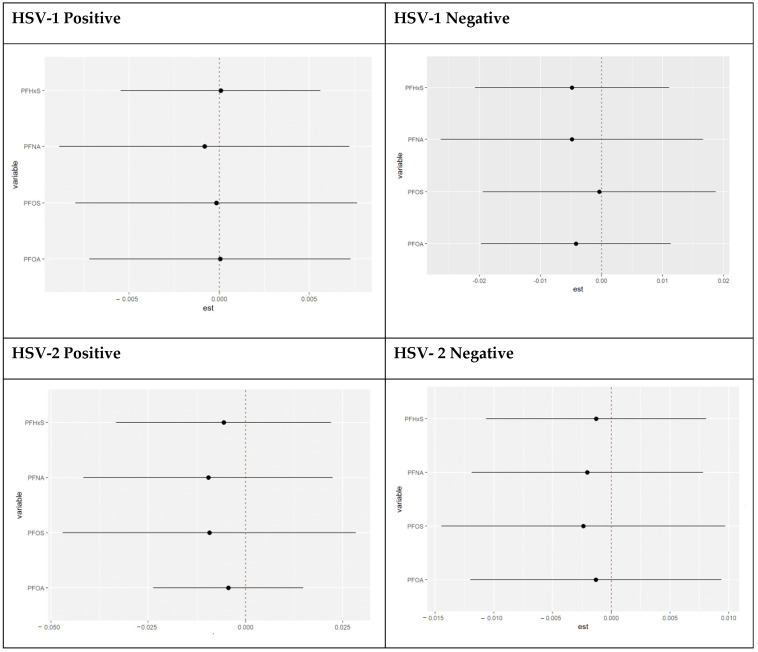
Chart showing the single variable interaction terms of PFOA, PFOS, PFNA, and PFNA at increasing quantiles of other PFAS variables.

This examines the interaction effects between PFAS exposures. It estimates the probability of the interaction’s inclusion for each pair of variables, indicating whether their joint effect significantly contributes to explaining the outcome variable beyond their main effects. Figure 8 provides a chart showing the overall effect of PFOA, PFOS, PFNA, and PFNA at increasing quantiles. This relates to subtracting the estimate represented by the red circle from the estimate represented by the blue circle in the above graph. The charts show the single-variable effect when the other PFAS components are fixed to their 25th quantile compared to when they are set at the 75th quantile.

## 4. Discussion

Researchers have found evidence suggesting that stress might trigger the reactivation of persistent infections [28,29]. In light of stress’s known role in negative health outcomes [9], it is crucial to include stress assessment in preventive strategies and treatments [14,15]. In this study, 60% of the participants tested positive for HSV-1, while 20% tested positive for HSV-2, indicating significant exposure to HSV among U.S. adults. As such, when considered in the context of PFAS exposure and AL, the study data were ideal for exploring our hypothesis.

Our study found a relationship between PFAS and AL among those exposed to HSV. This confirms previous studies, which indicated that most people may experience chronic stress [30], with this study being among the first to find that exposure to PFAS can potentially promote chronic stress, especially in the context of persistent infectious agents. Other studies have found that PFAS negatively affects the immune system [31,32], but little has been done to understand how that interacts with persistent infections. Indeed, our results revealed that participants not exposed to PFAS had a significant decrease of 77 to 97% in the odds of experiencing higher AL (*p* < 0.001).

Our findings, as revealed through descriptive statistics, also indicate that individuals testing positive for HSV-1 and HSV-2 are more likely to have elevated AL compared to those with non-elevated AL. In the study, 17.23% of those testing positive for HSV-1 exhibited higher AL, while 14.35% of those testing negative for HSV-1 had higher AL. Additionally, 17.17% of those testing positive for HSV-2 showed higher AL, in contrast to 14.35% of those testing negative for HSV-2. These outcomes collectively underscore the intricate relationship between chronic stress, PFAS exposure, and persistent infections.

Due to the study’s design, it remains unclear whether chronic stress leads to exposure to HSV or if exposure to HSV induces chronic stress. Nonetheless, it is evident that chronic stress can amplify pathogenic immune responses, hinder protective immunological responses, or both [33], making the former pathway more likely. Considering that both PFAS and chronic stress can weaken the immune system, it becomes more comprehensible why they might be interlinked in the presence of persistent infections. In cases where a relationship was observed even without our chosen persistent infection (HSV), it is plausible that the presence of other persistent infections or unexplored factors might have rendered the non-exposed category too broad to accurately signify non-exposure.

It should be noted that although the descriptive statistics showed that the mean PFAS values were similar for participants with and without higher AL, logistic regression showed patterns of association between PFAS and AL while accounting for confounders, complexity of the association, and non-linearity and distribution of the data [13]. Thus, these findings best represent the relationship, considering the comprehensiveness of the analysis technique. Previous studies have used logistic regression and recommended it as a better alternative to linear analytics techniques [13]. At the same time, researchers highlight the importance of using advanced statistical methods to report complex patterns among correlated components in environmental mixtures [26].

Adjusted models for participants testing positive for HSV-1 revealed a robust negative association with AL in the absence of exposure to PFOA, PFOS, PFNA, and PFHxS. These models demonstrated statistical significance (*p* < 0.05), underscoring the impact of PFOA, PFOS, PFNA, and PFHS on elevated AL. Furthermore, the null models highlighted that individuals not exposed to PFOA, PFOS, PFNA, and PFHxS exhibited odds reductions of 79% to 97% for experiencing higher AL. This signifies that exposure to PFOA, PFOS, PFNA, and PFHxS escalates the likelihood of higher AL, affirming previous research on the deleterious effects of PFAS exposure [12].

Beyond the elevation of AL, scientific investigations have linked PFAS exposure to heightened cholesterol levels, alterations in liver enzymes, diminished vaccine response, and increased risk of hypertension in pregnant women and cardiovascular ailments [2,4]. Given that AL serves as a potential mediator for various chronic diseases [13,34,35], it is plausible that the relationship between PFAS and AL, particularly in the context of persistent infections, extends to encompass broader implications. In this light, it can be hypothesized that in the presence of a persistent infection, those exposed to PFAS may experience chronic stress, which over extended periods could contribute to chronic conditions such as cardiovascular disease, liver disorders, and cancer, among others.

It may also demonstrate that even though HSV can persist over an extensive period in the body without causing much damage, exposures to environmental mixtures like PFAS are likely to reactivate the virus and cause additional stress, discomfort, and adverse health outcomes. Indeed, latent HSV-1 and HSV-2 are reactivated by stress-related stimuli, adrenal hormones, immunosuppression, and emotional disturbance [16], creating an environment for contaminants such as PFAS to contribute to disease processes. The biological persistence of PFAS [5] in the context of chronic stress paints a dire picture of the damage potentially caused by exposure to both over an extended period. This is a public health concern, and public health officials should continue with interventions to reduce the exposure of PFAS from the air, soil, water, manufacturing, and consumable products while incorporating stress prevention into all levels of health care.

Applying the logistic regression and BKMR models helped to understand the intricate non-linear and non-addictive association between PFAS and higher AL [26]. This underscores the importance of using BKMR to understand the extent of the association and interaction with PFAS components and AL. 

Previous studies have criticized the limitations of current statistical and machine learning methods like random forest for identifying essential mixtures rather than the direction of association [26]. The BKMR model sheds more light on the interactive effect of PFAS in the context of HSV and best models the impact of combined exposures.

The PIP for participants who tested positive for HSV-1 shows that even though PFHxS PIP: 0.4934 was more relevant, followed by PFOS PIP: 0.4700, PFOA PIP: 0.4659, and PFNA PIP: 0.4604, the combined relative importance of these PFAS on AL was similar. This speaks to the need to broadly focus on classes of chemicals when seeking to comprehensively protect at-risk populations from exposure and subsequent disease. A policy focusing on the PFAS exposome would prove to be most effective in mitigating the risk for vulnerable populations.

The study’s design and the employed statistical methods were instrumental in discerning the pivotal mixture component in relation to HSV-1 and HSV-2 exposure. Notably, there existed a marginal divergence in PIPs among the PFAS components for participants with HSV-1, which exhibited statistical significance compared to the other participant groups. This contrast illuminated a clear demarcation between the more impactful and less influential PFAS components for this subgroup. This variability in PIP values might be attributed to the prolonged effects of persistent infections on the body and how this uncontrolled viral presence could potentially interact with PFAS components, resulting in divergent health impacts, including heightened AL. It is important to note that prior studies have demonstrated that the protracted existence of persistent infections within infected cells leads to chronic conditions [16], which can have implications for AL. These findings align with previous research on the interactive effects of environmental mixtures on health [19,20,26,36,37,38].

The logistic regression, bivariate exposure-response association, interactive charts, and three-way interactive charts collectively confirm the presence of interactions between the components within this study. The PIPs further reveal that while PFOS and PFHxS were more critical among participants who tested positive for HSV-1 and HSV-2, the results indicate that PFOA, PFOS, PFNA, and PFHxS also influence AL levels. In this context, PFNA was found to be the least important among those with HSV-1 and HSV-2, whereas it played a significant role for those who tested negative for HSV-2. This indicates that the relative importance of PFAS may change as infectious status changes, underscoring the necessity to better comprehend the mechanistic underpinnings of varying degrees of infectious disease exposure levels.

The results demonstrated that participants without a college education were most likely to experience higher AL. As a result, the impact of these socioeconomic and demographic factors cannot be disregarded when studying the association between PFAS and AL. This confirms previous findings that demographic and social factors bring forth varied challenges that adversely affect health [6,8,10,19] and potentially speaks to the role PFAS and its interaction with demographic, socioeconomic, and environmental factors may intersect with structural inequality [8].

In both the logistic regression and BKMR models, the outcomes indicated that individuals with exposure to PFOS are more likely to have higher AL if they test positive for HSV-1, while those exposed to PFNA are less likely to experience higher AL in individuals testing positive for HSV-1. These associations underscore the synergistic relationship involving PFAS exposure, HSV-1 and HSV-2 status, and AL outcomes. The significance of adopting multiple statistical methodologies, such as logistic regression and BKMR, has been demonstrated in prior research [20]. The application of logistic regression and BKMR was pivotal in comprehending the interaction patterns of critical PFAS exposures and their association with elevated AL within the context of HSV-1 and HSV-2 test results. Researchers have effectively employed BKMR to investigate the collective impacts of environmental mixtures on a range of health outcomes [19,20].

The results of this study have significant implications for both public health and environmental policymakers. The study provides increased knowledge on the association between PFAS exposure, HSV infection, and AL. This information is necessary for implementing interventions to mitigate the health risks associated with PFAS exposure. The findings emphasize the need for continued research into the joint effect of environmental mixtures like PFAS and persistent infection on increased AL and quality of life. It highlights the impact of socioeconomic factors on increased stress and well-being.

### Limitations

One of the key limitations of this study is the use of cross-sectional data. Cross-sectional data provides a snapshot of information collected at a single point in time. While it is valuable for examining associations between variables at a specific moment, it cannot establish causality or capture changes over time. Longitudinal data, however, involves collecting information from the same individuals over multiple time points, allowing for the assessment of changes and trends over time.

Despite the limitations of using cross-sectional data, this study provides valuable insights into the association between multi-PFAS exposures and AL at a specific point in time. However, future research using longitudinal data is warranted to gain a more comprehensive understanding of the long-term effects of PFAS exposures and their implications for AL and overall health over time. Such insights could have important implications for public health interventions and policy decisions to reduce exposure to PFAS compounds and improve health outcomes for affected populations.

## 5. Conclusions

This study reveals significant associations between PFAS exposure and AL, considering HSV-1 and HSV-2 test results. Participants exposed to PFAS components showed increased odds of experiencing elevated AL. The results confirmed the hypotheses. All intercept values from the logistic regression model for participants exposed to PFAS stratified by HSV-1 and HSV-2 were significant, with *p* values of <0.001. This indicates that participants not exposed to PFAS have between 77% and 97% decreased odds of higher AL. This BKMR model provided an additional understanding of the extent of the association and its effect on AL. The results indicated that PFAS exposure may reactivate the association between HSV-1 and HSV-2 status and AL outcomes. These findings contribute to our understanding of the complex relationship between environmental exposures, infectious diseases, and physiological stress.

## Figures and Tables

**Table 1:** Characteristics of the sample participants.

Characteristics	Total Sample Population
N (%)	1784
Age: mean (SD)	33.62 (8.95)
PFOA: Mean (SD)	3.47 (3.32)
PFOS: Mean (SD)	11.08 (11.77)
PFNA: Mean (SD)	1.32 (1.36)
PFHxS: Mean (SD)	2.11 (2.73)
HSV-1	
Yes	60.59%
No	39.41%
HSV-2	
Yes	19.99%
No	80.01%
Gender	
Female	52.28%
Male	47.72%
Education	
Less than high school	22.86%
High school or equivalent	23.21%
Some college/associate degree	33.30%
Bachelor’s degree or higher	20.63%
Race/ethnicity	
Black	14.84%
White	57.08%
Hispanic	20.80%
Other	7.29%
Household Income	
<25,000	30.18%
25,000–<55,000	44.59%
55,000–<75,000	19.95%
75,000+	5.29%
Marital Status	
Married or Living with a partner	54.07%
Single	45.93%

**Table 2:** Characteristics of the sample participants stratified by AL status.

	Non-Elevated AL	Elevated AL	*p*-Value *
N (%)	1476 (82.7%)	307 (16.3%)	
Age: mean (SD)	32.95 (8.89)	37 (8.40)	
PFOA: Mean (SD)	3.50 (3.50)	3.28 (2.13)	0.7698
PFOS: Mean (SD)	10.98 (11.71)	11.56 (12.14)	0.5881
PFNA: Mean (SD)	1.34 (1.43)	1.22 (0.90)	0.5278
PFHxS: Mean (SD)	2.11 (2.64)	2.07 (3.14)	0.4129
HSV-1			
Yes	82.77%	17.23%	0.1507
No	85.65%	14.35%	
HSV-2			
Yes	82.83%	17.17%	0.6009
No	84.18%	15.82%	
Gender			
Female	81.02%	13.36%	0.0050
Male	86.72%	19.08%	
Education			
Less than high school	81.02%	18.98%	0.2121
High school or equivalent	84.03%	15.97%	
Some college/associate degree	83.60%	16.40%	
Bachelor’s degree or higher	87.48%	12.52%	
Race/ethnicity			
Black	84.12%	15.88%	0.9370
White	84.32%	15.68%	
Hispanic	82.77%	17.23%	
Other	83.50%	16.50%	
Household Income			
<25,000	82.67%	17.33%	0.3544
25,000–<55,000	83.06%	16.94%	
55,000–<75,000	86.05%	13.95%	
75,000+	90.10%	9.90%	
Marital Status			
Married or Living with a partner	81.82%	18.18%	0.0206
Single	86.37%	13.63%	

* The *p*-values were calculated from a Chi-Square test for categorical covariates and a Wilcoxon test for continuous covariates.

**Table 3:** Unadjusted logistic regression models examining the association between PFAS and AL stratified by HSV-1 and HSV-2 status.

Stratum	HSV-1 Negative		HSV-1 Positive		HSV-2 Negative		HSV-2 Positive	
Term	OR (95% CI)	*p*-Value	OR (95% CI)	*p*-Value	OR (95% CI)	*p*-Value	OR (95% CI)	*p*-Value
Intercept	0.23 (0.15, 0.37)	<0.0001	0.21 (0.18, 0.24)	<0.0001	0.21 (0.15, 0.28)	0	0.23 (0.14, 0.39)	0.0000
PFOS	1.02 (1.00, 1.05)	0.0349	–	–	1.02 (1.00, 1.04)	0.0900	0.96 (0.91, 1.02)	0.1630
PFNA	0.60 (0.42, 0.86)	0.0063	–	–	0.85 (0.73, 1.00)	0.0556	–	–
PFOA	–	–	–	–	–	–	–	–
PFHxS	–	–	–	–	0.95 (0.89, 1.01)	0.1087	1.15 (0.98, 1.35)	0.0870

**Table 4:** Covariate-adjusted logistic regression model examining the association between PFAS and AL among those exposed and unexposed to HSV.

Stratum	HSV-1 Negative		HSV-1 Positive		HSV-2 Negative		HSV-2 Positive	
Term	OR (95% CI)	*p*-Value	OR (95% CI)	*p*-Value	OR (95% CI)	*p*-Value	OR (95% CI)	*p*-Value
Intercept	0.23 (0.15, 0.37)	<0.0001	0.03 (0.01, 0.08)	<0.0001	0.21 (0.15, 0.28)	<0.0001	0.23 (0.14, 0.39)	<0.0001
Gender: Male	–	–	1.51 (1.07, 2.14)	0.0206	–	–	–	–
Age	–	–	1.06 (1.03, 1.08)	<0.0001	–	–	–	–
HH Income: 25,000–<55,000	–	–	0.81 (0.52, 1.27)	0.3432	–	–	–	–
HH Income: 55,000–<75,000	–	–	0.59 (0.33, 1.05)	0.0724	–	–	–	–
HH Income: 75,000+	–	–	0.40 (0.18, 0.91)	0.0287	–	–	–	–
Marital Status: Single	–	–	0.54 (0.34, 0.85)	0.0085	–	–	–	–
PFOS	1.02 (1.00, 1.05)	0.0350	–	–	1.02 (1.00, 1.04)	0.0900	0.96 (0.91, 1.02)	0.1630
PFNA	0.60 (0.42, 0.86)	0.0063	–	–	0.85 (0.73, 1.00)	0.0556	1.15 (0.98, 1.35)	0.0870
PFHxS	–	–	–	–	0.95 (0.89, 1.01)	0.1087	–	–

**Table 5:** Logistic regression model examining the association between PFAS and AL and accounting for the interaction between various PFAS components.

Stratum	HSV-1 Negative		HSV-1 Positive		HSV-2 Negative		HSV-2 Positive	
Term	OR (95% CI)	*p*-Value	OR (95% CI)	*p*-Value	OR (95% CI)	*p*-Value	OR (95% CI)	*p*-Value
Intercept	0.23 (0.12, 0.37)	<0.0001	0.03 (0.01, 0.08)	<0.0001	0.21 (0.15, 0.28)	<0.00001	0.23 (0.14, 0.39)	<0.0001
PFOS	1.02 (1.00, 1.05)	0.03495	1.04 (0.96, 1.14)	0.0206	1.02 (1.00, 1.04)	0.0900	0.96 (0.91, 1.02)	0.163
PFOA	–	–	1.01 (0.78, 1.32)	<0.0001	–		–	–
PFNA	0.60 (0.42, 0.86)	0.00634	1.22 (0.71, 2.10)	0.3432	0.85 (0.73, 1.00)	0.0556	–	–
PFHxS	–	–	0.56 (0.32, 0.99)	0.0724	0.95 (0.89, 1.01)	0.1087	1.15 (0.98, 1.35)	0.0870
PFOS: PFOA	–	–	1.00 (0.98, 1.01)	0.7401	–	–	–	–
PFOS: PFNA	–	–	0.97 (0.94, 1.00)	0.0389	–	–	–	–
PFOA: PFNA			0.91 (0.77, 1.07)	0.2251	–	–		
PFOS: PFHxS	–	–	1.02 (1.00, 1.03)	0.0643	–	–	–	–
PFOA: PFHxS	–	–	1.07 (0.97, 1.18)	0.1713	–	–	–	–
PFNA: PFHxS	–	–	1.17 (0.84, 1.64)	0.3408	–	–	–	–
PFOS:PFOA: PFNA	–	–	1.00 (1.00, 1.01)	0.0344	–	–	–	–
PFOS:PFOA: PFHxS	–	–	1.00 (0.99, 1.00)	0.1469	–	–	–	–
PFOS:PFNA: PFHxS	–	–	1.00 (0.99, 1.00)	0.3540	–	–	–	–
PFOA:PFNA: PFHxS	–	–	1.00 (0.94, 1.06)	0.9149	–	–	–	–
Gender: Male	–	–	1.61 (1.02, 2.54)	0.0411	–	–	–	–
Age	–	–	1.06 (1.04, 1.09)	<0.00001	–	–	–	–
HH Income: 25,000–<55,000	–	–	0.80 (0.51, 1.26)	0.3270	–	–	–	–
HH Income: 55,000–<75,000	–	–	0.58 (0.32, 1.04)	0.0685	–	–	–	–
HH Income: 75,000+	–	–	0.40 (0.17, 0.92)	0.0334	–	–	–	–
Marital Status: Single	–	–	0.55 (0.35, 0.88)	0.0138	–	–	–	–

**Table 6:** PIP values from the BKMR models stratified by participants’ test results for HSV-1 and HSV-2.

	HSV-1		HSV-2	
	Positive	Negative	Positive	Negative
PFOA	0.4659	0.3305	0.4408	0.2970
PFOS	0.4700	0.4060	0.4526	0.4732
PFNA	0.4604	0.4853	0.3435	0.4553
PFHxS	0.4934	0.3486	0.4473	0.36072

## Data Availability

The NHANES dataset is publicly available online, accessible at cdc.gov/nchs/nhanes/index.htm (accessed on 5 February 2023).
